# Effect of Ionomer on CO_2_ Reduction at Atomically Dispersed Ni─N─C Catalyst

**DOI:** 10.1002/smll.202511445

**Published:** 2025-10-19

**Authors:** Youngdon Ko, Hengquan Guo, Luigi Osmieri, Hanguang Zhang, Piotr Połczyński, Santosh Adhikari, Seung Geol Lee, Yu Seung Kim, Piotr Zelenay

**Affiliations:** ^1^ Materials Physics and Applications Division Los Alamos National Laboratory Los Alamos NM 87545 USA; ^2^ Department of Materials Science and Engineering Ulsan National Institute of Science and Technology (UNIST) Ulsan 44919 Republic of Korea; ^3^ Chemical Diagnostics and Engineering Group Los Alamos National Laboratory Los Alamos NM 87545 USA; ^4^ Graduate School of Semiconductor Materials and Devices Engineering Ulsan National Institute of Science and Technology (UNIST) Ulsan 44919 Republic of Korea

**Keywords:** carbon dioxide reduction, CO_2_RR, catalyst‐ionomer interactions, ionomer, Ni‐N‐C catalyst

## Abstract

Ionomers play an important role in carbon dioxide (CO_2_) electrolyzers by affecting CO_2_ reduction reaction (CO_2_RR) at the cathode. In this study, we systematically examines the impact that different ionomers have on CO_2_RR at atomically dispersed nickel–nitrogen–carbon (Ni─N─C) catalyst. We compare the effects of two acidic cation exchange ionomers, perfluorosulfonic acid (Nafion) and sulfonated poly(aryl ether sulfone), and two alkaline anion exchange ionomers: quaternary ammonium‐functionalized poly(aryl piperidinium) and quaternary ammonium‐functionalized polyfluorene. Of the four ionomers, the Nafion‐bonded electrode reaches the highest CO_2_RR activity and selectivity in H‐cell testing likely due to the polymer weak interaction with the Ni─N─C catalyst. Flow cell measurements further validate superior CO_2_RR performance of the Nafion‐bonded electrode relative to those containing other polymers. The findings from this research highlight a critical importance of interactions between the ionomers and the catalyst surface in CO_2_ reduction, which go beyond their well‐established role in regulating local pH and hydrophobic/hydrophilic properties of the catalyst layer.

## Graphical abstract

Ionomer properties play a considerable role in tuning activity and selectivity of catalysts for electrochemical CO_2_ reduction reaction (CO_2_RR). This work reports on the effect of two cation exchange and two anion exchange ionomers on the performance of an atomically dispersed Ni─N─C electrocatalyst for CO_2_ reduction to CO. The results highlight significance of the ionomer selection for maximizing CO_2_RR performance of cathode catalysts in CO_2_ electrolyzers.

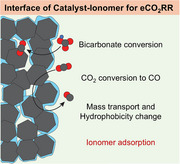

## 1 Introduction

The electrochemical carbon dioxide reduction reaction (CO_2_RR) has the potential to help address critical energy and environmental challenges by offering a clean path to conversion of atmospheric CO_2_ to value‐added products such as fuels and chemicals. Of various CO_2_RR products, CO has attracted considerable attention as: i) CO_2_ conversion to CO requires only two‐electron and two‐proton transfer, thus making it the simplest CO_2_RR pathway; ii) CO is an important intermediate for the synthesis of C_2+_ products;^[^ ^1^, ^2^ ^]^ iii) CO is widely used as a reducing agent in industries such as metallurgical, pharmaceutical, and electronics;^[^ ^3^ ^]^ and iv) it can be readily converted into high‐value chemicals via thermocatalytic processes.^[^ ^4^ ^]^

Numerous studies have shown that ionomer‐coated catalysts effectively facilitate CO production via CO_2_RR over a wide range of potentials.^[^ ^5^, ^6^, ^7^, ^8^, ^9^, ^10^, ^11^, ^12^, ^13^ ^]^ However, while the impact of ionomers on CO_2_RR activity is well recognized, the question of which ionomer performs best remains unresolved. Liu et al. compared the performance of Nafion® and Fumion for CO_2_‐to‐CO conversion when combined with an Ag catalyst, finding that while both ionomers exhibited similar performance in H‐cells, Nafion was less effective in zero‐gap cells.^[^ ^14^ ^]^ Wang et al. investigated CO_2_RR activity of an Ni─N─C catalyst combined with various anion exchange ionomers in KHCO_3_ electrolytes. Their findings suggest that all anion exchange ionomers perform similarly in the kinetic region, but achieving high current densities requires a suitable balance between anion conductivity and ionomer hydrophobicity. They also hypothesized that cationic exchange headgroups in Nafion could create a bipolar junction, thereby hindering ion transfer and reducing electrolyzer performance.^[^ ^15^ ^]^ Yu et al. investigated the effects of Nafion and anion exchange ionomers on Ag and cobalt phthalocyanine (CoPc) supported on nanocarbon catalysts for CO_2_RR. Their results show that the CoPc catalyst exhibits higher CO_2_RR activity when bonded with Nafion, compared to anion exchange ionomers.^[^ ^16^ ^]^ These findings point to inconsistencies in the literature when it comes to the effect of ionomers on CO_2_RR. While some studies have explored the pH effects of ionomers on CO_2_RR, other ionomer‐related impacts have remained insufficiently examined.^[^ ^17^, ^18^, ^19^ ^]^

In this work, we focus on the influence of ionomers on the CO_2_RR activity of an atomically dispersed Ni─N─C catalyst. The synthesis, composition, and performance details of the Ni─N─C catalysts through high‐temperature treatment of an Ni‐doped zeolitic imidazole framework (ZIF‐8) have been described previously.^[^ ^20^, ^21^ ^]^ Here, the synthesis method was scaled up, with the Ni‐to‐Zn of 1:10 ratio selected to assure atomic dispersion of the Ni─N─C sites in the catalyst (catalyst previously referred to as Ni_1.0%_‐N‐C^[^ ^21^ ^]^). Following the synthesis, the absence of Ni nanoparticles was verified by X‐ray diffraction (XRD), transmission electron microscopy (TEM), and scanning electron microscopy (SEM) (Figures S1 and S2, Supporting Information).

**Figure 1:**
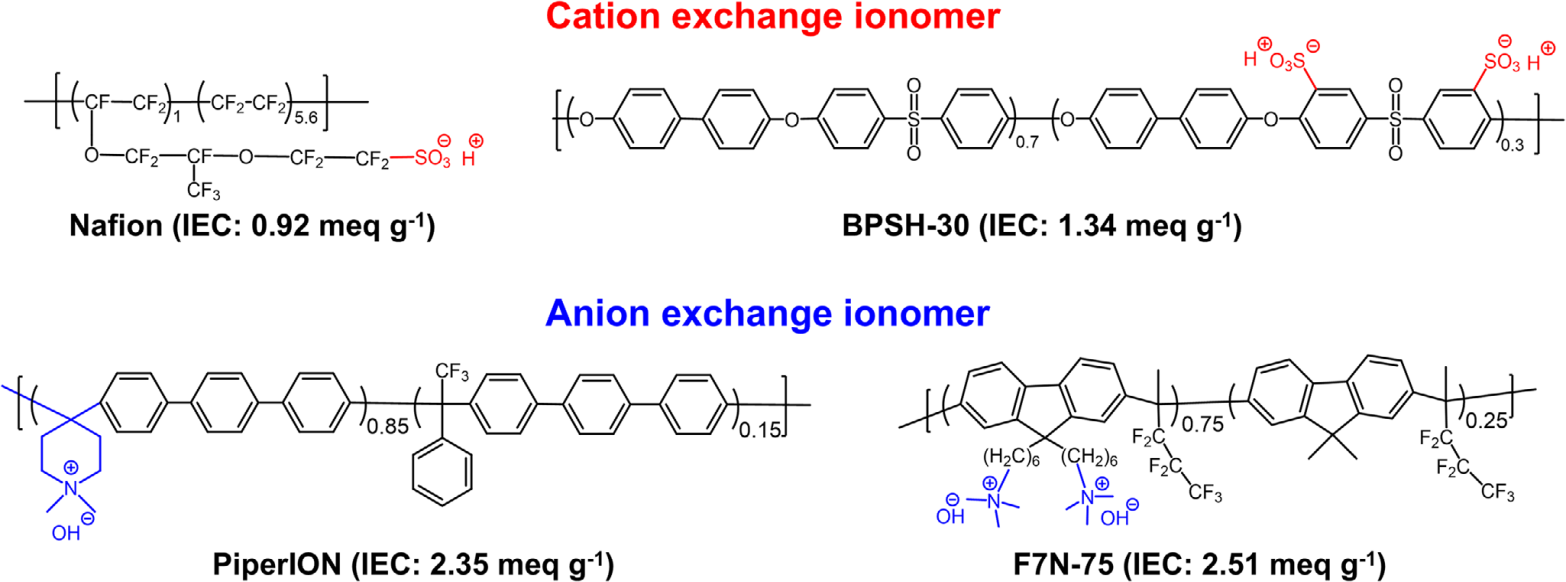
Chemical structure and IEC values of ionomers used in this study: Nafion, BPSH‐30, PiperION, and F7N‐75.

For this study, we selected two acidic cation exchange ionomers, perfluorosulfonic acid (Nafion) and sulfonated poly(arylene ether sulfone) (BPSH‐30),^[^ ^22^ ^]^ and two alkaline anion exchange ionomers: commercial quaternary ammonium‐functionalized poly(piperidinium) (PiperION®)^[^ ^23^ ^]^ and quaternary ammonium‐functionalized poly(fluorene) (F7N‐75).^[^ ^24^ ^]^ The chemical structure and ion exchange capacity (IEC) of these ionomers are shown in **Figure** **1**.

CO_2_RR activity was evaluated using different electrochemical setups: an H‐cell and a flow cell, both operated with KHCO_3_ electrolytes.

## 2 Results and Discussion

### 2.1 Impact of Ionomers on Bicarbonate Conversion in H‐Cell

While externally delivered CO_2_ is a majority reagent to form CO, some CO can be also generated from bicarbonate (KHCO_3_) through the following Equations ((1), (2), (3)):

(1) $$KHCO_{3}↔\;K^{+}+HCO_{3}^{-}$$

(2) $$HCO_{3}^{-}+3H^{+}+2e^{-}\to CO+2H_{2}O\;\;\;\;\;\;\;\;\;\;\;\;\left(\mathrm{acidic}\;\mathrm{condition}\right)$$

(3) $$HCO_{3}^{-}+H_{2}O+2e^{-}\to CO+3OH^{-}\;\;\;\left(\mathrm{neutral}\;\mathrm{or}\;\mathrm{alkaline}\;\mathrm{condition}\right)$$

**Figure 2:**
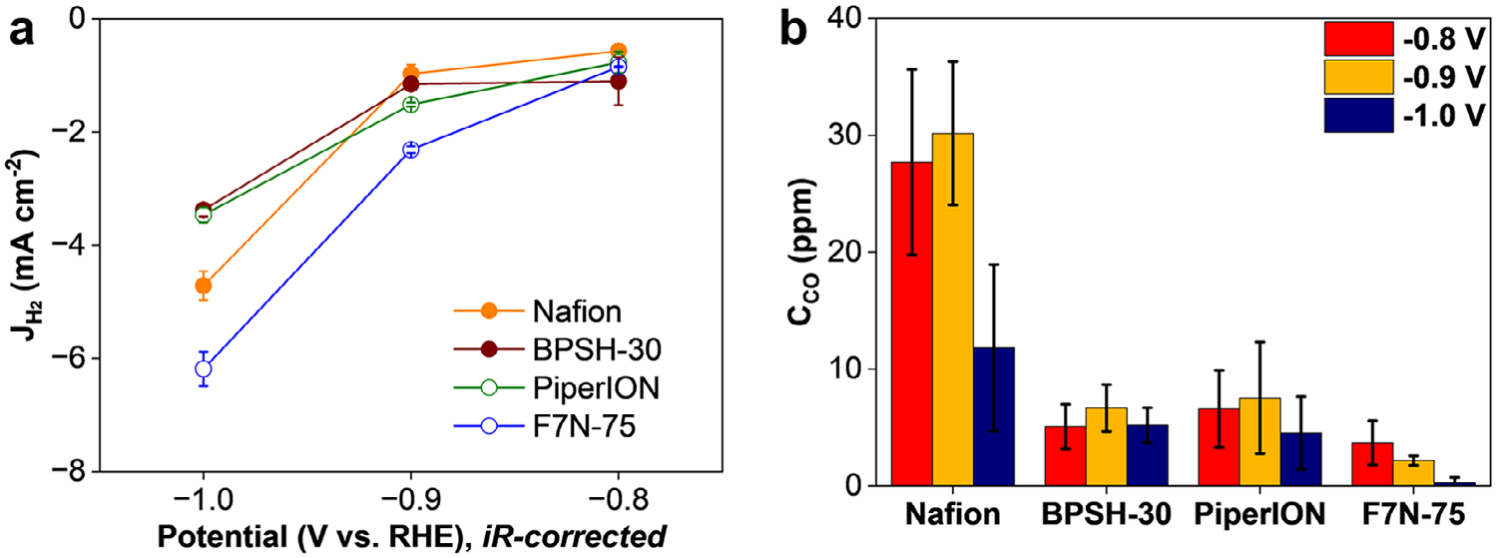
Bicarbonate conversion to CO at electrodes containing different ionomers in Ar‐purged 0.1 M KHCO_3_ (H‐cell): a) HER current density and b) CO production at different electrode potentials.

The CO production from bicarbonate was measured in this work using an H‐cell in 0.1 M KHCO_3_ electrolyte purged with ultra‐high purity Ar (99.999%). The hydrogen evolution reaction (HER) current density from water splitting at −0.8 V and −0.9 V was notably higher with F7N‐75‐ and Nafion‐bonded electrodes (**Figure** **2**a) than the other two electrodes. While faradaic efficiency of bicarbonate conversion was the highest with Nafion‐bonded electrode, it was still less than 5%, thus attesting to negligible CO production (Figure 2b). At the same time, the differences in bicarbonate conversion rates by as much as a factor of five show that the reaction is affected by the ionomer choice.

### 2.2 Impact of Ionomers on CO_2_RR Performance in H‐Cell

**Figure 3:**
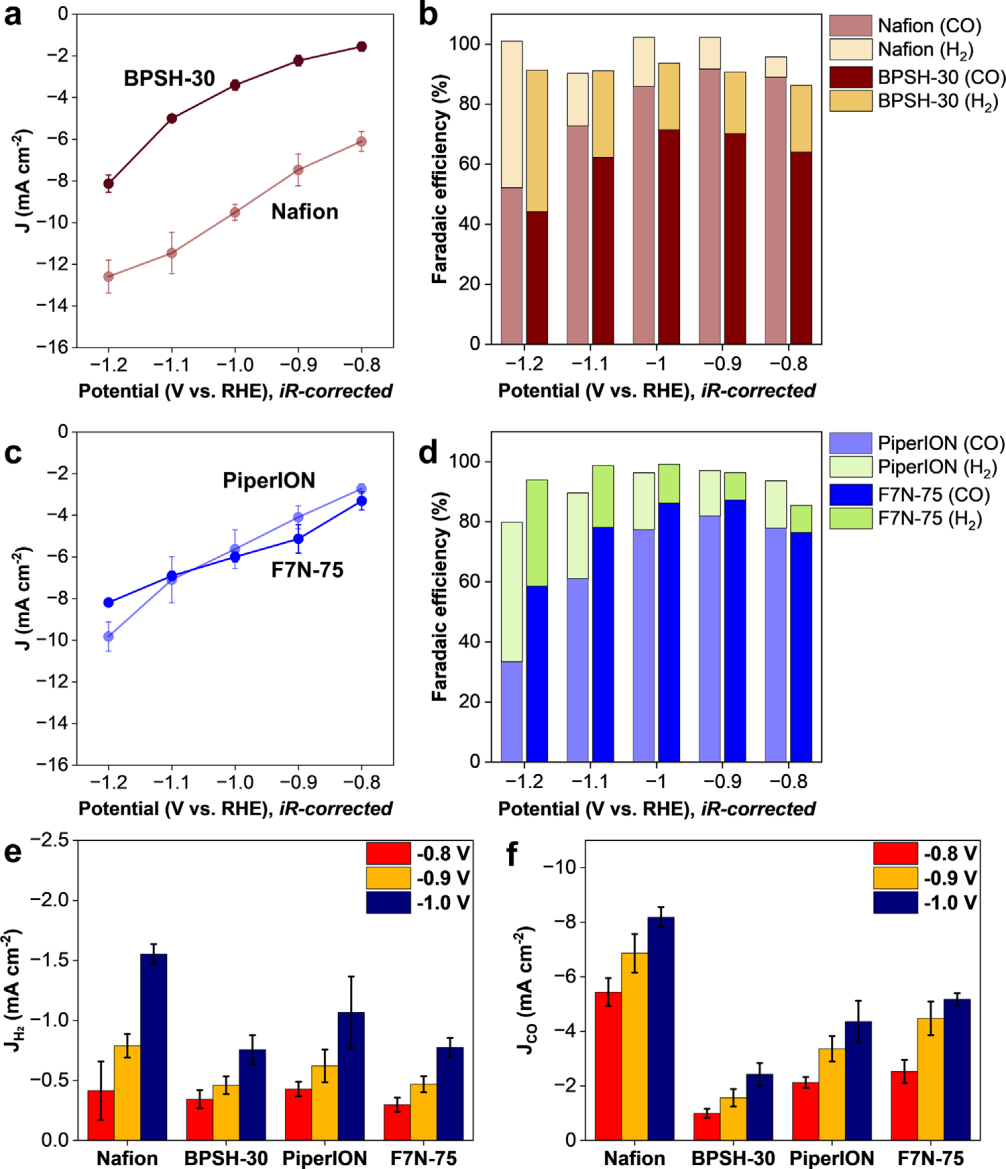
CO_2_RR performance of Ni─N─C catalyst with different ionomer‐bonded electrodes in an H‐cell: a) total current density and b) faradaic efficiency of Nafion‐ and BPSH‐30‐bonded electrodes; c) total current density and d) faradaic efficiency of PiperION‐ and F7N‐75‐bonded electrodes; e) partial HER current density measured with four electrodes; f) partial CO current density for four electrodes. Measurements conducted in an H‐cell with 1 cm^2^ electrode (4 mg cm^−2^ Ni─N─C catalyst loading) in a 0.1 m KHCO_3_ electrolyte using a CO_2_ flow rate of 15 mL min^−1^.

**Figure** **3** summarizes CO_2_RR performance of Ni─N─C catalyst in different ionomer‐bonded electrodes in an H‐cell. Of the two cases involving cation‐exchange ionomers (Figure 3a,b), a significantly higher total current density was recorded with the Nafion‐bonded than BPSH‐30‐bonded electrode. Also higher with Nafion‐bonded electrode was the faradaic efficiency for CO, 91.8% at −0.9 V, compared to 70.2% for the BPSH‐30‐bonded electrode. Nafion is more hydrophobic than BPSH‐30, as confirmed by the contact angle values of 130.9° and 27.8°, respectively (Figure S3a, Supporting Information), which is expected to facilitate gas diffusion.

The total current density values recorded with anion‐exchange ionomers, PiperION and F7N‐75 (Figure 3c,d), were comparable, however, faradaic efficiency for CO at −0.9 V was slightly higher with F7N‐75‐bonded electrode than the PiperION‐bonded one (87.3% and 82.0%, respectively). The F7N‐75‐bonded electrode was also more hydrophobic (contact angle value of 109.1° vs. 67.4° for PiperION, Figure S3b, Supporting Information), expected to facilitate CO_2_ transport.

The partial HER current density decreased significantly with all electrodes when CO_2_ was supplied instead of Ar. The partial current density for hydrogen evolution was reduced by ≈2.2 times at −0.8 V and by as much as 4.3 times at −1.0 V. Of the four systems, the Nafion‐bonded electrode generated the highest current density for hydrogen generation, followed by the PiperION‐bonded electrode (Figure 3e).

Figure 3f summarizes partial CO current density measured with four electrodes at different potentials. Of the four, the Nafion‐bonded electrode generated the highest CO_2_RR current density and the BPSH‐30‐bonded one the lowest at all studied potentials. CO_2_RR performance of two anion exchange systems, PiperION and F7N‐75, falls between those of the two electrodes prepared with acidic ionomers. These results suggest that the pK_a_ value of the ionomer is not the primary factor that determines CO_2_RR activity, as supporting electrolytes present in large excess in the system neutralize the sulfonic acid groups in acidic ionomers. Instead, the presented results point to another factor that determines the CO_2_RR activity, which is not directly associated with the CO_2_ as a reagent: the ionomer‐catalyst surface interactions.

### 2.3 DFT Study of Ionomer Adsorption on the Ni─N─C Catalyst

To gain insight into the catalyst‐ionomer interactions, the adsorption energy of four fragments in Nafion, BPSH‐30, PiperION, and F7N‐75 ionomers most likely to interact with the Ni─N─C catalyst surface was calculated by density functional theory (DFT). The selected ionomer fragments were most prone to adsorb on the Ni─N─C catalyst and represent the key structural features of the ionomer. They are depicted in Figure S5 (Supporting Information). Following approach used in prior studies,^[^ ^25^, ^26^ ^]^ the Ni─N─C catalyst surface was modelled as a defect‐free carbon monolayer featuring nitrogen atoms coordinated with a central nickel atom. Molecular fragments were placed directly atop the Ni site for adsorption. For each fragment, two distinct initial adsorption orientations were considered. In the “horizontal” configuration, the phenyl ring or carbon backbone is oriented parallel to the Ni─N─C surface. Conversely, in the “vertical” configuration, the phenyl ring or carbon backbone is oriented perpendicular to the Ni─N─C surface, as illustrated in Figure S6 (Supporting Information).

**Figure 4:**
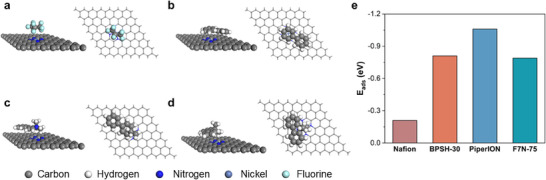
Most stable DFT‐optimized adsorption configurations of representative molecular fragments in a) Nafion, b) BPSH‐30, c) PiperION, and d) F7N‐75 ionomers on the Ni─N─C surface. For each structure, the left panel shows a tilted perspective view, and the right panel displays the corresponding top view to clearly depict the adsorption orientation. e) Calculated adsorption energy values for the four fragments.

After full optimization, the horizontal configuration was found to interact more strongly than the vertical configuration for the selected groups in BPSH‐30, PiperION, and F7N‐75, thus representing the most stable adsorption cases. In contrast, in Nafion, marginally stronger adsorption (by only 0.01 eV) was determined for the vertical orientation. This subtle energy difference can be attributed to the high structural symmetry inherent to the Nafion fragment. All adsorption energy values are provided in Table S1 (Supporting Information), with corresponding configurations and depicted in **Figure** **4**a–d. The adsorption energy of the PiperION in the horizontal configuration is the most negative, −1.06 eV, indicating the strongest interaction with the Ni─N─C catalyst surface. BPSH‐30 and F7N‐75 in horizontal configuration come next with similar adsorption energy values of −0.81 and −0.79 eV, respectively (Figure 4e). The interaction of Nafion in the parallel configuration is the weakest based on the calculated adsorption energy of −0.21 eV. These findings point to PiperION as interacting more strongly with the Ni─N─C catalyst than BPSH‐30 and F7N‐75, and Nafion being the least prone to adsorb on the catalyst surface.

### 2.4 Ionomer Impact on CO_2_RR Durability

**Figure 5:**
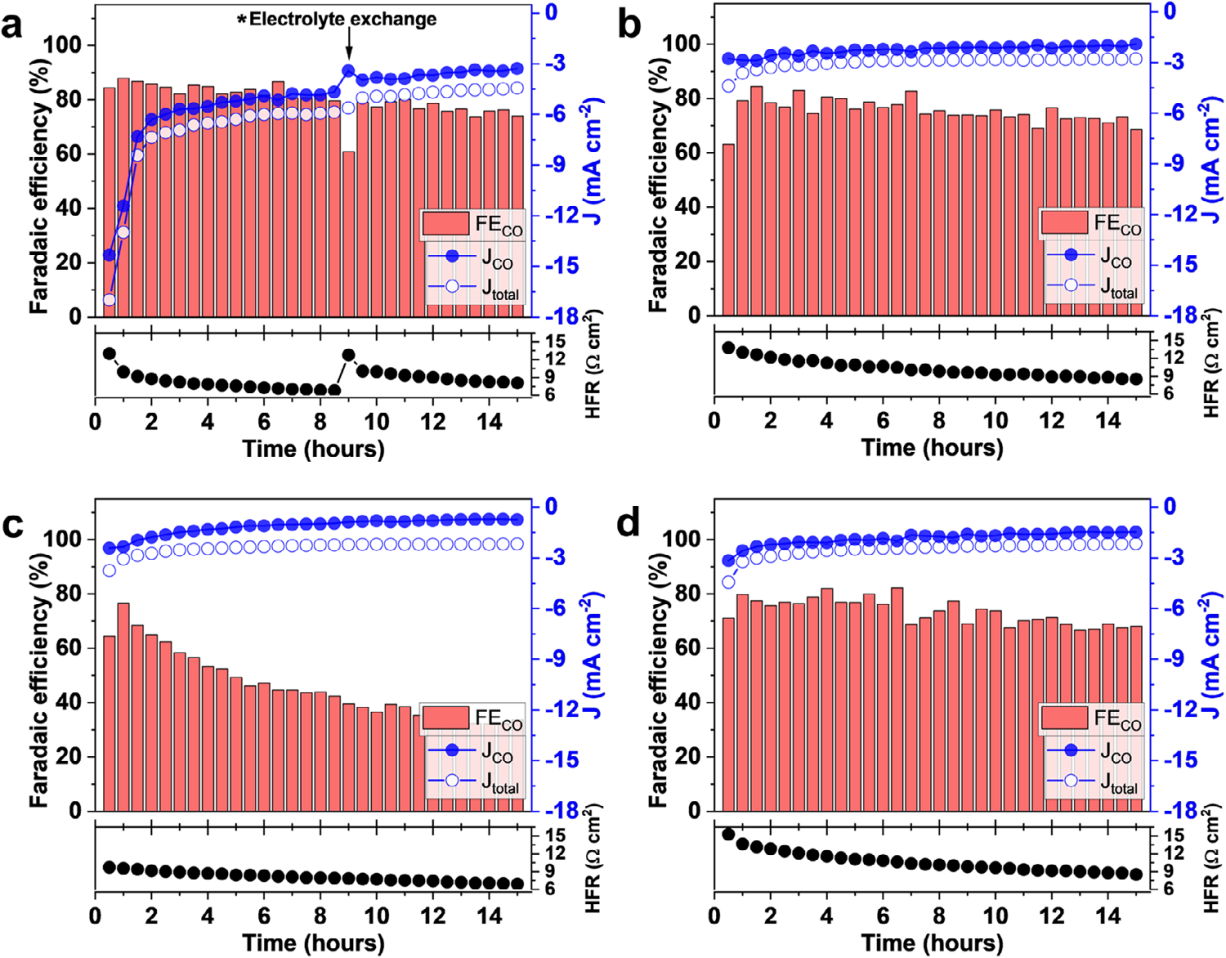
Faradaic efficiency and current density for different ionomer‐bonded Ni─N─C electrodes as a function of time at −1.0 V vs. RHE a) Nafion, b) BPSH‐30, c) PiperION, and d) F7N‐75. Measurements conducted in an H‐cell using 1 cm^2^ electrode (4 mg cm^−2^ Ni─N─C catalyst loading) in 0.1 m KHCO_3_ electrolyte purged with CO_2_ at a flow rate of 15 mL min^−1^.

Short durability tests performed in an H‐cell show a gradual performance decrease with all four ionomers during the first ca. 70 min (**Figure** **5**). The Nafion‐bonded electrode experienced the most significant current loss, likely a combined effect of the proton‐potassium ion exchange, CO_2_RR and HER in 0.1 M KHCO_3_. To minimize the ion exchange during reaction, Nafion ionomer was neutralized (protons in Nafion sulfonic groups exchanged with potassium ions) in 1.0 M KOH prior to the electrode fabrication. As a result, the initial performance loss was reduced by approximately a factor of two compared to the performance loss of the cell operating with a protonated form of Nafion (Figure S7, Supporting Information). The performance of protonated form of Nafion‐bonded electrode stabilized faster in 0.5 M KHCO_3_ electrolyte (Figure S8a, Supporting Information, potential −1.0 V *vs*. RHE), thanks to higher potassium ion concentration helping faster equilibration. However, at a higher potential of −0.9 V, i.e., at lower current densities, the current stabilization occurred more gradually (Figure S9, Supporting Information).

A steady decrease in the high‐frequency resistance (HFR) was observed during the durability testing. This was attributed to changes in the combined effect of CO_2_RR, HER, and potassium ion transport, resulting in increased solution conductivity. The replacement of the electrolyte after 9 h of cell operation brought the HFR back to its original value (Figure 5a, Nafion‐bonded electrode). Notwithstanding this conductivity improvement, the faradaic efficiency and partial CO current density slowly decreased with time of cell operation.

Faradaic efficiency for CO generation with the PiperION‐bonded electrode was much lower than in the other three cases. While the commonly observed decrease in faradaic efficiency was likely related to the local pH change at the catalyst‐ionomer interface, the generally low faradaic efficiency measured with the PiperION‐bonded electrode might have been caused by cation‐hydroxide co‐chemisorption on the catalyst surface.^[^ ^27^ ^]^

### 2.5 CO_2_RR Performance Validation in Flow Cell

The CO_2_RR current density in the H‐cell is limited by low solution concentration of CO_2_ (ca. 33 mm in 0.1 m KHCO_3_^[^ ^28^ ^]^). To validate the ionomer effect in more practical systems, we evaluated CO_2_RR activity in a flow cell, where gaseous CO_2_ was directly supplied to the carbon‐fiber side of the gas diffusion electrode and electrolyte was flown across the Ni─N─C catalyst‐coated side of the microporous layer.

**Figure 6:**
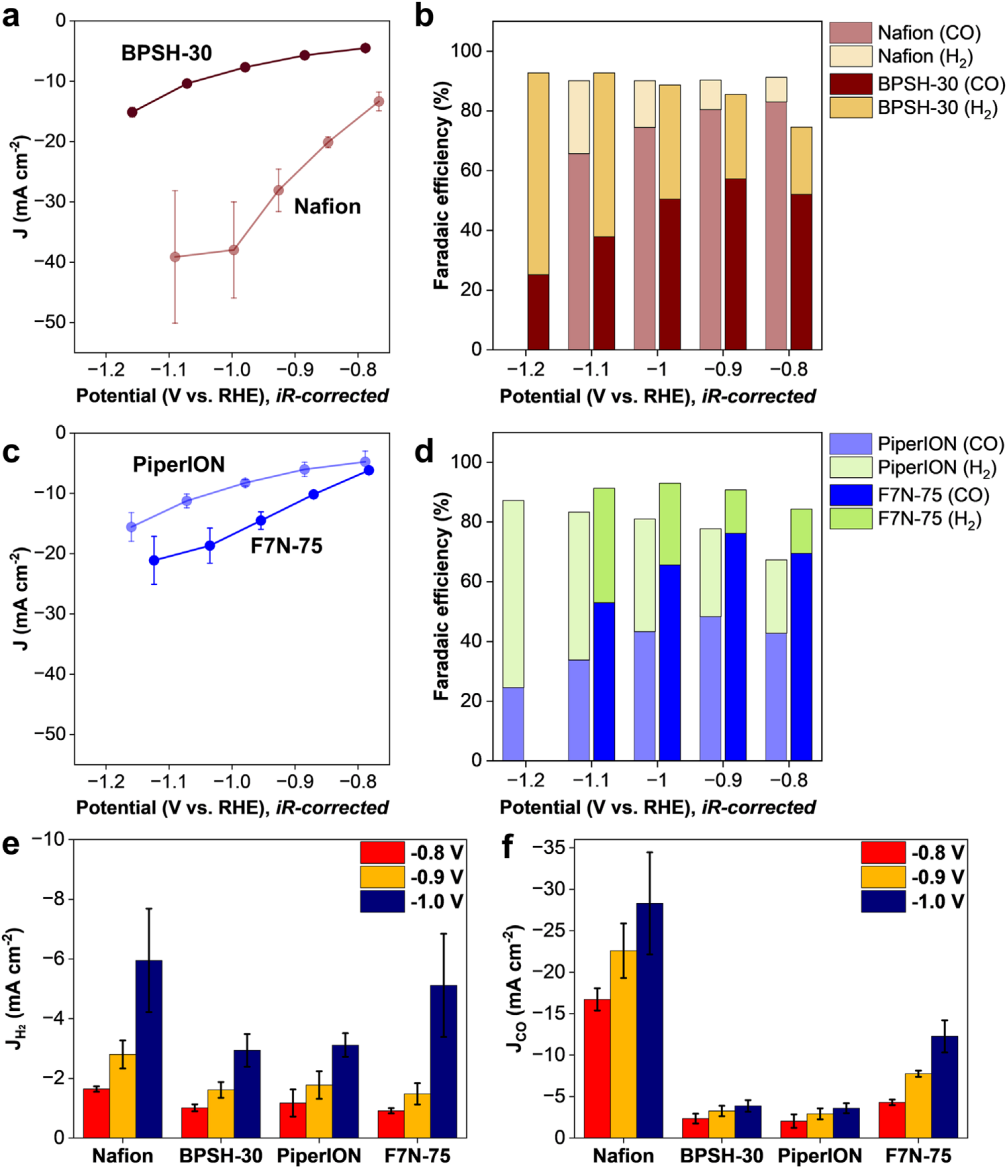
CO_2_RR performance of Ni─N─C catalyst with different ionomers in a flow cell: a) total current density and b) faradaic efficiency for Nafion‐ and BPSH‐30‐bonded electrodes; c) total current density and d) faradaic efficiency for PiperION‐ and F7N‐75‐bonded electrodes; e) partial HER current density values; f) partial CO current density values. Cathode catalyst loading 4 cm^2^ electrode, CO_2_ flow 30 mL min^−1^, 1 m KHCO_3_ flow 10 mL min^−1^ (anode and cathode).

CO_2_RR activity and selectivity in a flow cell operating with different ionomer‐bonded electrodes are summarized in **Figure** **6**. Of the four systems, the highest current density across the entire cathode potential range was obtained with the Nafion‐bonded electrode. Partial HER current densities were very similar for all ionomers, except Nafion, in which case a slightly higher HER activity was determined. At −1.0 V vs. RHE, the partial CO current density values measured with different ionomers were as follows: Nafion – 28.3 mA cm^−2^; BPSH‐30 – 3.9 mA cm^−2^; PiperION – 3.6 mA cm^−2^; F7N‐75 – 12.3 mA cm^−2^. The Nafion‐bonded electrode was also the most effective at achieving high CO_2_RR activity and selectivity, in agreement with the H‐cell results (Figure 3).

When tested without a catalyst the carbon substrates showed considerable HER activity and low CO faradaic efficiency. The H‐cell results obtained with pristine and ionomer‐coated carbon papers in Figures S10 and S11 (Supporting Information) confirm considerable contribution of the carbon support to the HER, especially at high overpotentials, with partial HER current densities comparable to the ones measured with not yet catalyzed, Nafion‐ and BPSH‐30‐coated substrates. By contrast, the electrodes coated with PiperION and F7N‐75 ionomers showed lower HER activity than the carbon‐paper support alone, attesting to differences in the ionomer interactions with the carbon. When the Ni─N─C catalyst was incorporated into the ionomers, the HER activity was significantly suppressed relative to that of the bare and ionomer‐coated carbon substrates. This suggests that Ni─N─C is largely CO_2_RR‐selective, while HER predominantly occurs on exposed carbon fibers, not coated with either the catalyst or the ionomer. These findings highlight a detrimental role of the carbon substrate in catalyzing HER, an important factor to consider when assessing CO_2_RR selectivity, especially at high overpotentials.

## 3 Conclusion

In this work, the effect of ionomer on the activity and selectivity of atomically dispersed Ni─N─C CO_2_RR catalyst was systematically evaluated. In an Ar‐purged H‐cell, the bicarbonate conversion measurements revealed that of the four studied ionomer‐bonded systems the highest bicarbonate conversion to CO occurs at the Nafion‐bonded electrode.

In a CO_2_‐purged H‐cell, the Nafion‐bonded electrode also exhibited the best performance. This can be attributed to the advantages of the Nafion ionomer, including its high hydrophobicity and relatively weak interaction with the Ni─N─C catalyst. DFT simulations confirmed that Nafion was the least prone to adsorb on the catalyst surface, with the calculated adsorption energy of −0.21 eV, by a factor of between 3.8 and 5.2‐fold less negative than adsorption energy values calculated for the hydrocarbon‐based ionomers. These differences in adsorption strength likely impact the availability of active sites and thus the overall catalyst performance.

The CO_2_RR activity trends observed in the H‐cell were validated in the flow cell. In agreement with the H‐cell data, the Nafion‐bonded electrode outperformed electrodes containing other ionomers. These findings underscore the critical role of ionomer selection in tuning interfacial catalyst–ionomer interactions to maximize the activity and selectivity of CO_2_RR catalysts.

## 4 Experimental Section

### Chemicals and Materials

The following chemicals were purchased from Sigma‐Aldrich and used without further purification: zinc nitrate hexahydrate (99%), nickel nitrate hexahydrate (97%), 2‐methylimidazole (97%), potassium bicarbonate (99.7%), potassium hydroxide (85%), methanol (99.9%), 2‐propanol (99.9%), dimethylacetamide (98%), and n‐hexane (95%). The PiperION‐A5‐HCO3EtOH ionomer (5 wt.% dispersion in ethanol) and PiperION‐A0‐HCO3 membrane (60 µm thickness) were purchased from Versogen. Carbon paper (Sigracet, SGL 39BB) was purchased from Fuel Cell Store. Nafion D521 ionomer (Chemours, 5wt.%) and Nafion 115 membrane (Chemours, 127 µm) were purchased from Ion Power. Platinized Ti felt was purchased from Giner. Deionized water (DI) was obtained using a Millipore Milli‐Q system (resistivity: 18.2 MΩ cm). The synthesis methods of BPSH‐30 and F7N‐75 ionomers were described in previous reports.^[^ ^22^, ^24^ ^]^ The BPSH‐30 membrane was dissolved in dimethylacetamide to prepare a 5 wt.% BPSH‐30 ionomer. The neutralized Nafion was prepared by titrating Nafion D521 ionomer with 1.0 M potassium hydroxide, adjusted according to the IEC value.

### Synthesis of ZIF‐8

The synthesis of ZIF‐8 was a scaled‐up version of the method reported previously.^[^ ^21^ ^]^ 45 g of zinc nitrate hexahydrate and 50 g of 2‐methylimidazole were separately dissolved in 300 mL of methanol. The solutions were mixed in a 1.0 L conical flask and stirred at 50 °C for 24 h to form a white precipitate. This precipitate was then centrifuged at 8,000 rpm and washed three times with methanol before being dried under vacuum at 50 °C overnight to obtain a white ZIF‐8 powder.

### Synthesis of Ni─N─C Catalyst

0.5 g of ground ZIF‐8 powder was placed into a 50 mL glass vial and dispersed in 20 mL of n‐hexane under constant mixing. Four vials were individually prepared. The container was sealed to inhibit evaporation of n‐hexane. In a separate vial, 1 g of nickel nitrate hexahydrate was dissolved in 10 mL of deionized water. 262 µL of the nickel nitrate solution was added to the ZIF‐8 dispersion in ten equal portions of 26.2 µL each, maintaining an atomic ratio of 1/10 of Ni‐to‐Zn in the precursor (Ni‐ZIF‐8). The Ni‐ZIF‐8 dispersion was further stirred for 2 h and then filtered under vacuum. The powder was subsequently collected and dried under a vacuum at 50 °C for 6 h, resulting in the modification of the powder color from white to light green. The Ni‐ZIF‐8 powder was crushed into a fine powder and then pyrolyzed for 2 h at 1000 °C under Ar flow using a 1‐hour ramp‐up time to produce the Ni─N─C electrocatalyst. The quantity of Ni‐ZIF‐8 powder used prior to pyrolysis was approximately 2 g. After the pyrolysis, ≈0.6 g of Ni─N─C catalyst powder was obtained.

### Materials Characterization

SEM images were acquired using a Thermo Fisher Scientific Teneo SEM. TEM images were obtained with a Thermo Fisher Scientific Titan 200 kV TEM. XRD spectra were collected on a Siemens D5000 diffractometer with Cu K_α_ radiation (λ = 1.54 Å) and a graphite monochromator. The diffraction patterns were recorded in the 2θ range from 20° to 80° with a step size of 0.1°. Water contact angle measurements were conducted using a Ramé‐Hart Model 500 Goniometer.

### Electrode Preparation

CO_2_RR activity and selectivity measurements with different ionomers were conducted after optimizing the catalyst and ionomer loading. The catalyst ink was prepared by dispersing the required amount of as‐synthesized Ni─N─C catalyst powder with ionomer in a 3:1 volumetric ratio of 2‐propanol and water. The catalyst ink was sonicated for at least 20 min in a water bath and then brush‐painted onto the microporous side of SGL 39BB carbon paper. The catalyst loading and the ionomer‐to‐catalyst (I/C) mass ratio were optimized using Nafion (Figures S12–S14, Supporting Information). The I/C ratio optimization demonstrated an increase in electrochemical activity with a decrease in the I/C ratio from 0.8 to 0.3, which was attributed to improved accessibility of the catalyst active sites. However, catalyst detachment from carbon paper occurred when the I/C ratio was less than 0.3. After the optimization of the loading and I/C ratio, the Ni─N─C catalyst loading was 4 mg cm^−2^ and I/C mass ratio was fixed at 0.3 for H‐cell testing. The catalyst loading was optimized at 7 mg cm^−2^ with the same I/C ratio of 0.3 for the flow‐cell testing. The electrode morphology with different ionomers was measured by SEM (Figure S4,Supporting Information). The catalyst was uniformly distributed on the electrode, with no significant morphological differences observed with all four electrodes. The electrodes for testing the ionomer‐coated carbon paper were prepared with the same ionomer composition.

### Electrochemical CO_2_ Reduction Testing

The electrochemical cell was an H‐shaped, gas‐tight cell consisting of two compartments: one for the oxygen evolution reaction and one for the CO_2_RR. Nafion 115 membrane was used to separate the two compartments. The cathode compartment included a leak‐free Ag/AgCl reference electrode (eDAQ) and a working electrode (catalyst coated on carbon paper substrate with a geometric area of 1 cm^2^). CO_2_ was continuously bubbled into the 0.1 M KHCO_3_ catholyte both before and during the CO_2_RR at a flow rate of 15 mL min^−1^. Pt mesh served as the counter electrode in the anode compartment. CO_2_RR measurements were performed after 10 voltammetric cycles between 0 and −1.2 V vs. RHE, 50 mV s^−1^. All cathode potential values were iR‐corrected. In the case of the 15‐hour potential‐hold experiments in the H‐cell, the potential values were iR‐corrected using HFR (high‐frequency resistance) values measured every 30 min.

A 4‐cm^2^ CO_2_RR flow cell was purchased from DEK Research Instrumentation. The Hg/HgO reference electrode was placed in the catholyte chamber. 1 m KHCO_3_ was used as an anolyte and catholyte and flown at a flow rate of 10 mL min^−1^ on both sides of the cell. The CO_2_ flow rate was kept at 15 mL min^−1^. IrO_2_ catalyst supported on Ni foam was used as counter electrode and 60‐µm thick PiperION membrane was used to separate the anode and cathode compartments. All cathode potential values were iR‐corrected. To calculate the faradaic efficiency in both the H‐cell and the flow‐cell experiments, the cathode potential was held constant for a total of 46 min: 20 min for stabilization and 26 min for two online gas chromatography measurements. The error bars were calculated from at least two independent CO_2_RR tests. The error bars can be larger in cases where smaller amounts of gas were generated, due to the low current density of the measured electrode.

### DFT Calculations of Ionomer Adsorption Energy

To predict the adsorption behavior of Nafion, BPSH‐30, PiperION, and F7N‐75 at the Ni─N─C catalyst, spin‐polarized density functional theory (DFT) calculations were performed using the Vienna Ab initio Simulation Package (VASP).^[^ ^29^, ^30^ ^]^ The interaction between core and valence electrons was described using the projector augmented wave (PAW) method,^[^ ^31^, ^32^ ^]^ while the exchange‐correlation effects were treated within the generalized gradient approximation (GGA) employing the Perdew–Burke–Ernzerhof (PBE) functional.^[^ ^33^ ^]^ Van der Waals interactions were accounted for via DFT‐D3 dispersion correction.^[^ ^34^ ^]^ A plane‐wave energy cutoff of 450 eV and a 3 × 3 × 1 Monkhorst‐Pack k‐point grid were employed.^[^ ^35^ ^]^ To eliminate spurious interlayer interactions, a vacuum layer of 15 Å was introduced along the c‐axis. The convergence criteria were set to 10^−5^ eV for electronic energy and 0.01 eV ^Å−1^ for atomic forces.

The adsorption energy (*E_ads_*) of molecules was calculated using the following Equation (4):^[^ ^36^, ^37^ ^]^

(4) $$E_{\mathrm{ads}}=E_{\mathrm{total}}-E_{\mathrm{molecule}}-E_{\mathrm{sub}}$$

where *E_total_* denotes the total energy of the molecule adsorbed on the Ni─N─C surface, *E_molecule_* represents the total energy of the molecule in vacuum, and *E_sub_* corresponds to the energy of the pristine Ni─N─C catalyst.

## Conflict of Interest

The authors declare no conflict of interest.

## Supporting information



Supporting Information

## Data Availability

The data that support the findings of this study are available from the corresponding author upon reasonable request.
