# Supplementary material for: Effect of Ionomer on CO2 Reduction at Atomically Dispersed Ni─N─C Catalyst
Source: Small. 2025 Oct 19;21(49):e11445. doi: 10.1002/smll.202511445 (PMC12696775; doi:10.1002/smll.202511445)
Supplement: Supplementary file 1 — Supporting Information [file SMLL-21-e11445-s001.docx]

Supporting Information

Effect of Ionomer on CO_2_ Reduction at Atomically Dispersed Ni-N-C Catalyst

Youngdon Ko,^a^ Hengquan Guo,^b^ Luigi Osmieri,^a^ Hanguang Zhang,^a^ Piotr Połczyński,^a^ Santosh Adhikari,^c^ Seung Geol Lee,^b,d^ Yu Seung Kim,^a^ Piotr Zelenay ^a^*

^a^ *Materials Physics and Applications Division, Los Alamos National Laboratory, Los Alamos, NM 87545, USA*

*^b^ Department of Materials Science and Engineering, Ulsan National Institute of Science and Technology (UNIST), Ulsan 44919, Republic of Korea*

*^c^ Chemical Diagnostics and Engineering Group, Los Alamos National Laboratory, Los Alamos, NM 87545, USA*

*^d^ Graduate School of Semiconductor Materials and Devices Engineering, Ulsan National Institute of Science and Technology (UNIST), Ulsan 44919, Republic of Korea*

*E-mail*: zelenay@lanl.gov


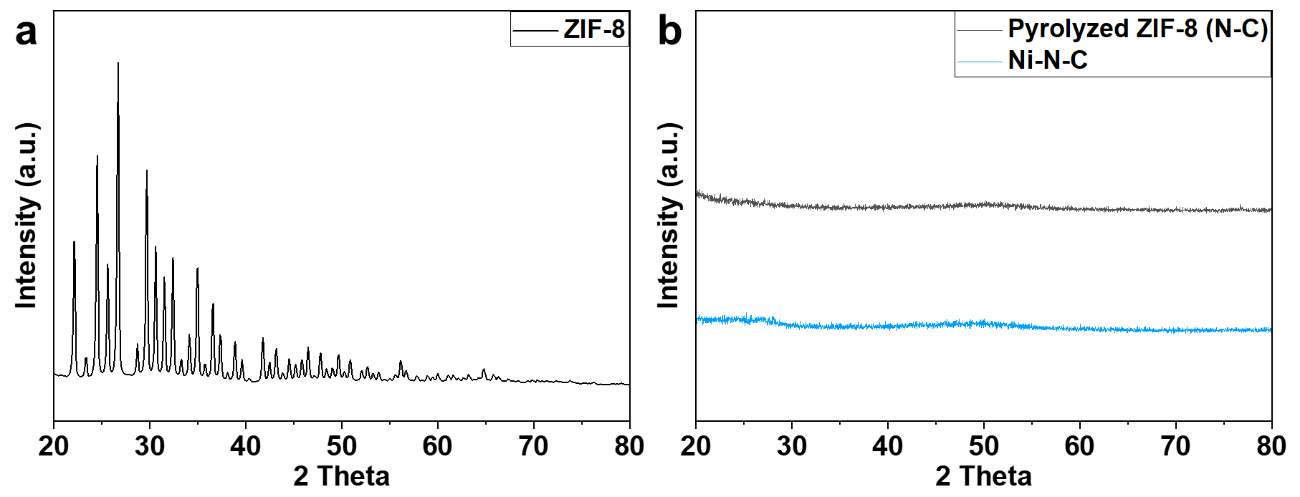


**Figure S1.** XRD pattern of (**a**) as-synthesized ZIF-8 and (**b**) N-C and Ni-N-C catalysts.


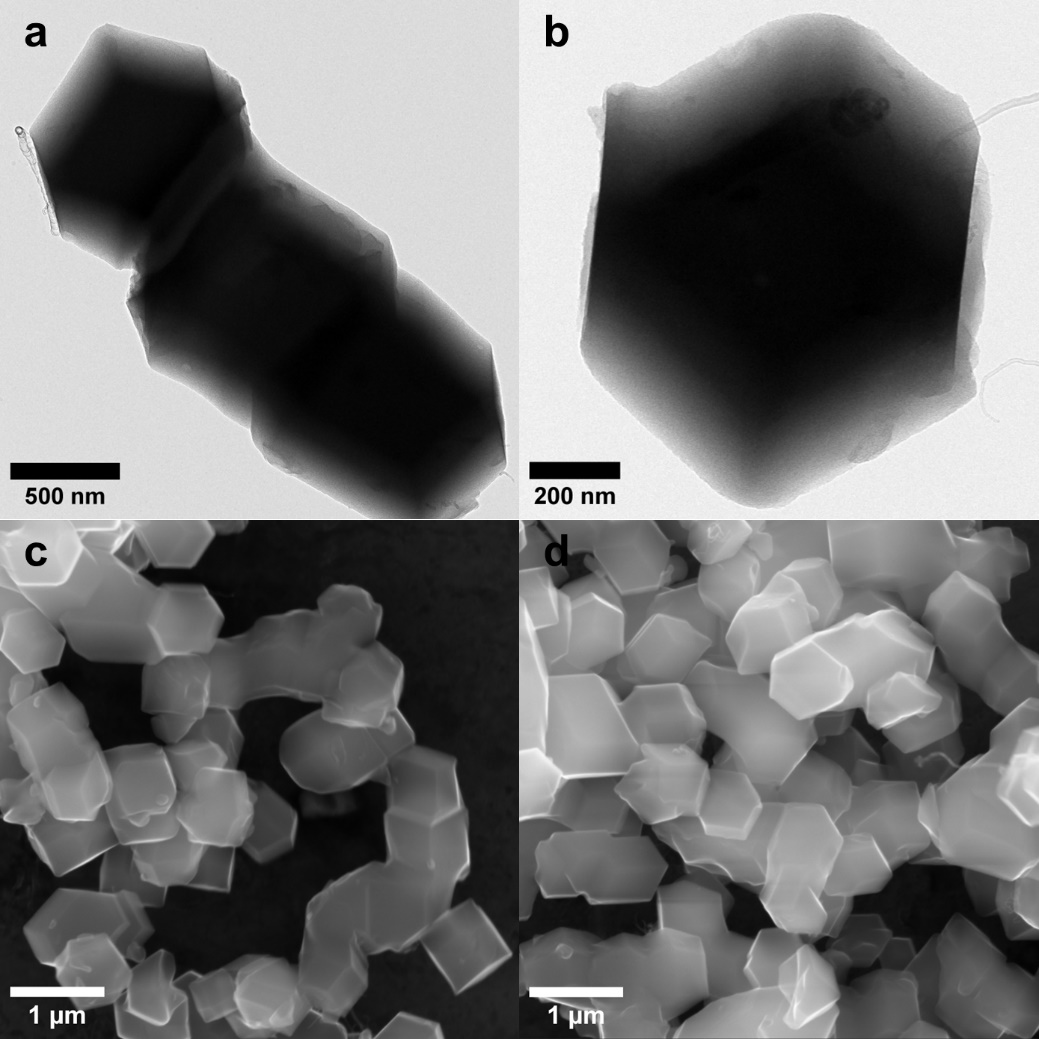


**Figure S2.** (**a**, **b**) TEM and (**c**, **d**) SEM images of Ni-N-C catalyst.


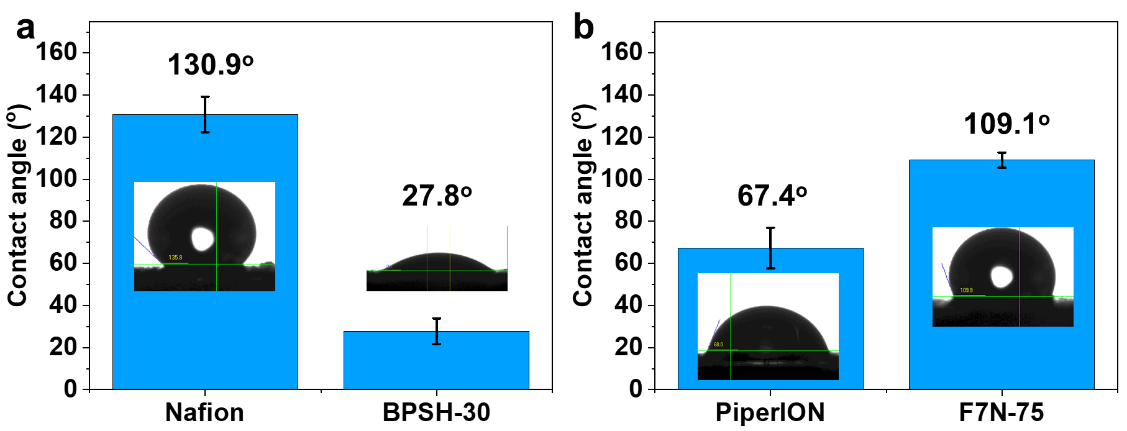


**Figure S3.** Water contact angle measurements at Ni-N-C catalyst with (**a**) cation exchange ionomers and (**b**) anion exchange ionomers deposited onto SGL 39BB carbon paper.


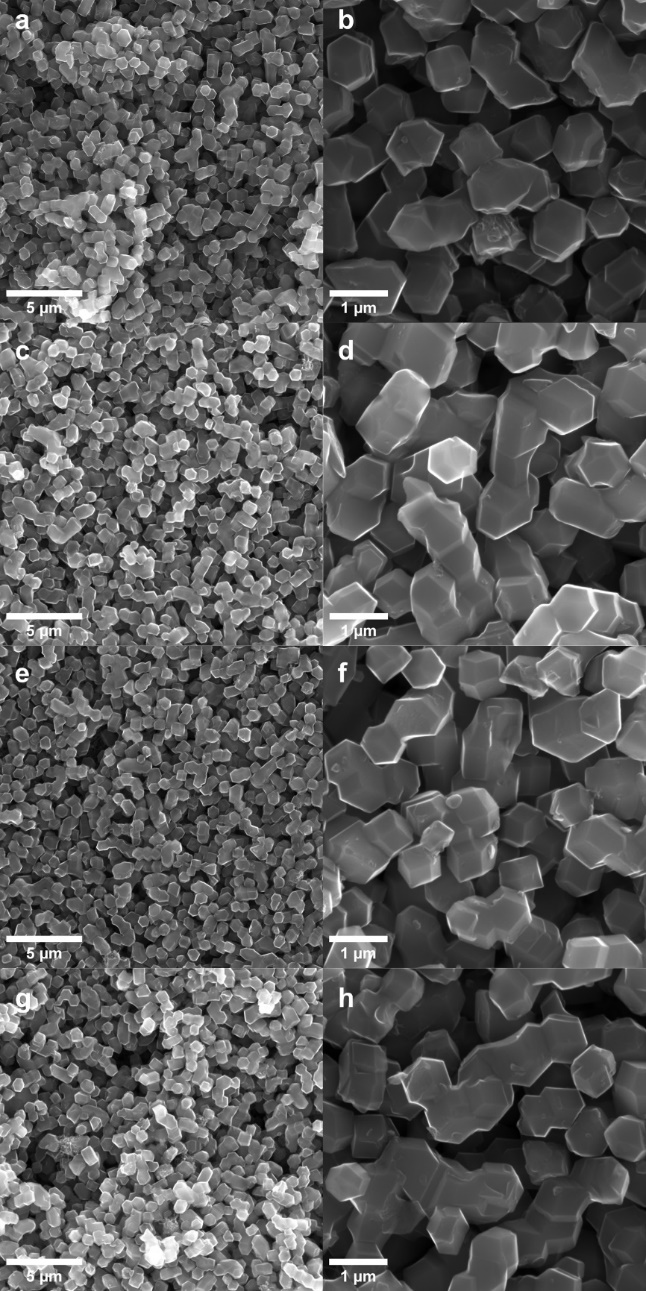


**Figure S4.** SEM images of Ni-N-C catalyst with ionomer deposited on SGL 39BB carbon paper with different ionomers: (**a, b**) Nafion, (**c, d**) BPSH-30, (**e, f**) PiperION, and (**g, h**) F7N-75.


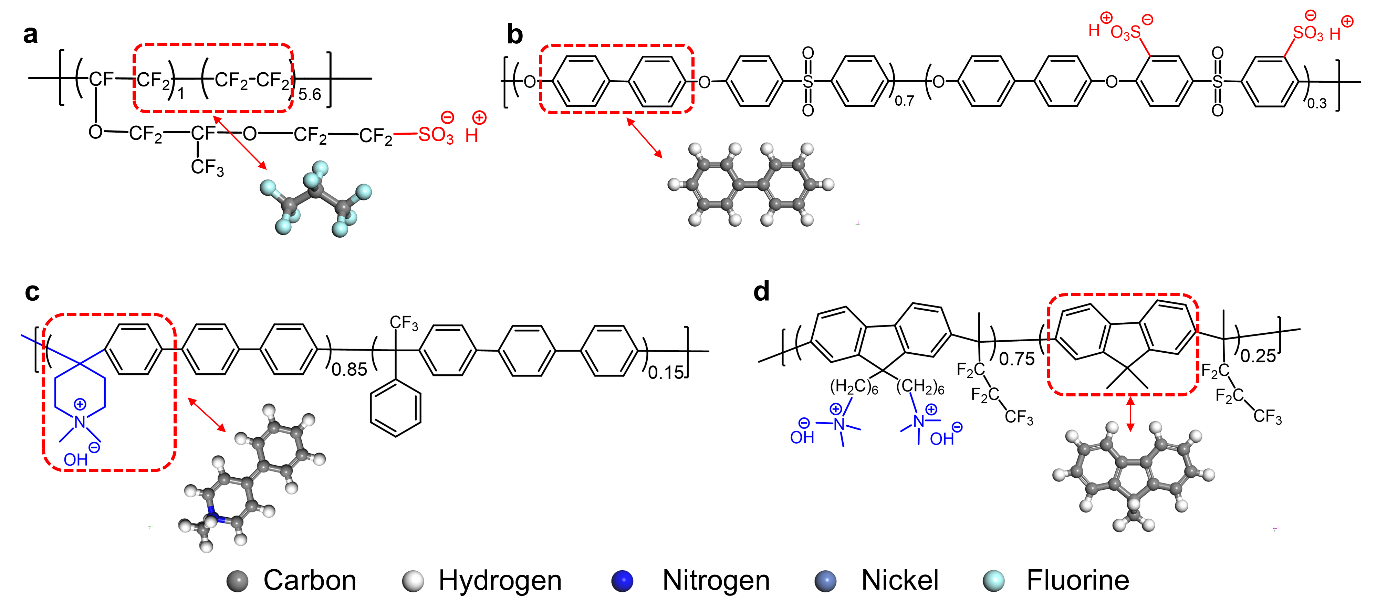


**Figure S5**. Molecular fragments (highlighted in red dashed boxes) selected to model the adsorption behavior of ionomers on the Ni-N-C catalyst surface in DFT calculations: (**a**) Nafion, (**b**) BPSH-30, (**c**) PiperION, and (**d**) F7N-75. The optimized structures of these fragments are shown in ball-and-stick models below their corresponding chemical structures.


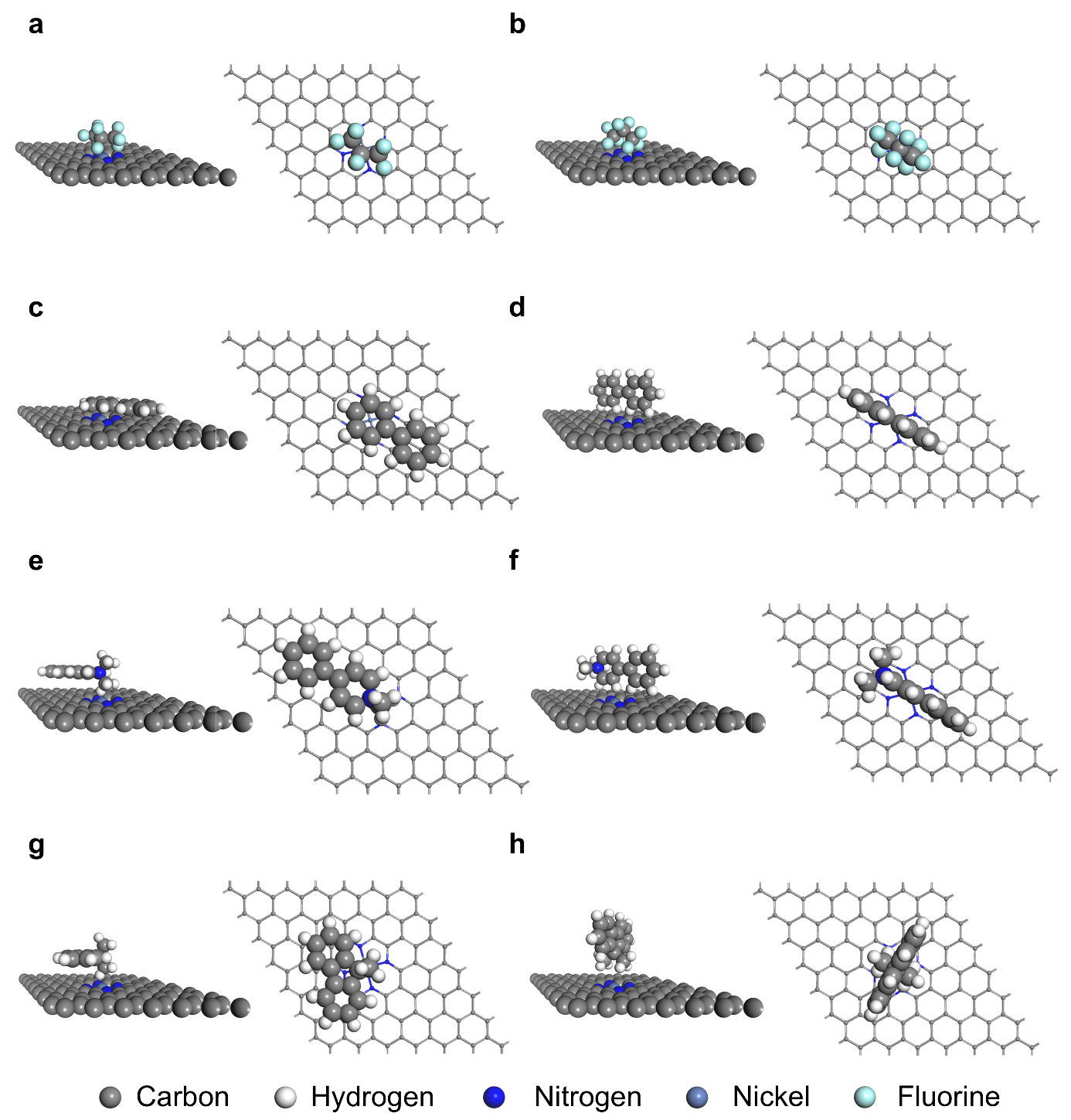


**Figure S6**. Adsorption configurations of molecular fragments derived from (**a, b**) Nafion, (**c, d**) BPSH-30, (**e, f**) PiperION, and (**g, h**) F7N-75 ionomers on the Ni-N-C surface. Each configuration is shown in tilted perspective (left) and top view (right). Panels (**a**), (**c**), (**e**), and (**g**) depict horizontal adsorption, while panels (**b**), (**d**), (**f**), and (**h**) illustrate vertical adsorption configurations.


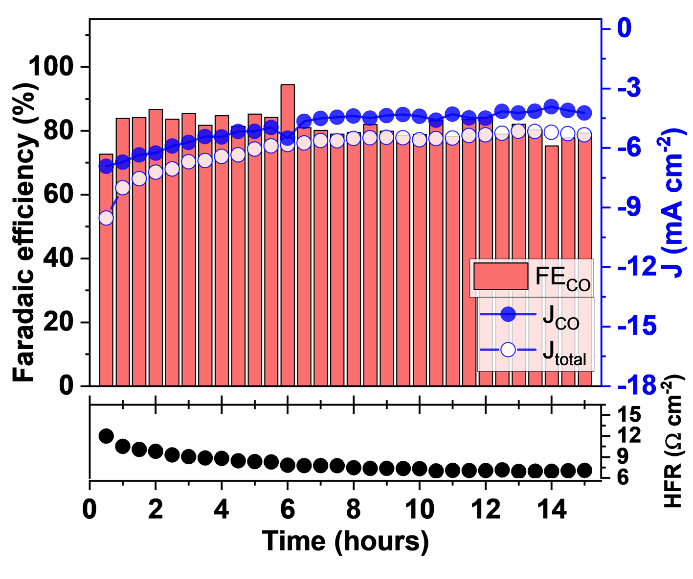
**Figure S7.** Durability test of Ni-N-C catalyst in a neutralized Nafion-bonded electrode at ‑1.0 V vs. RHE in 0.1 M KHCO_3_.

**
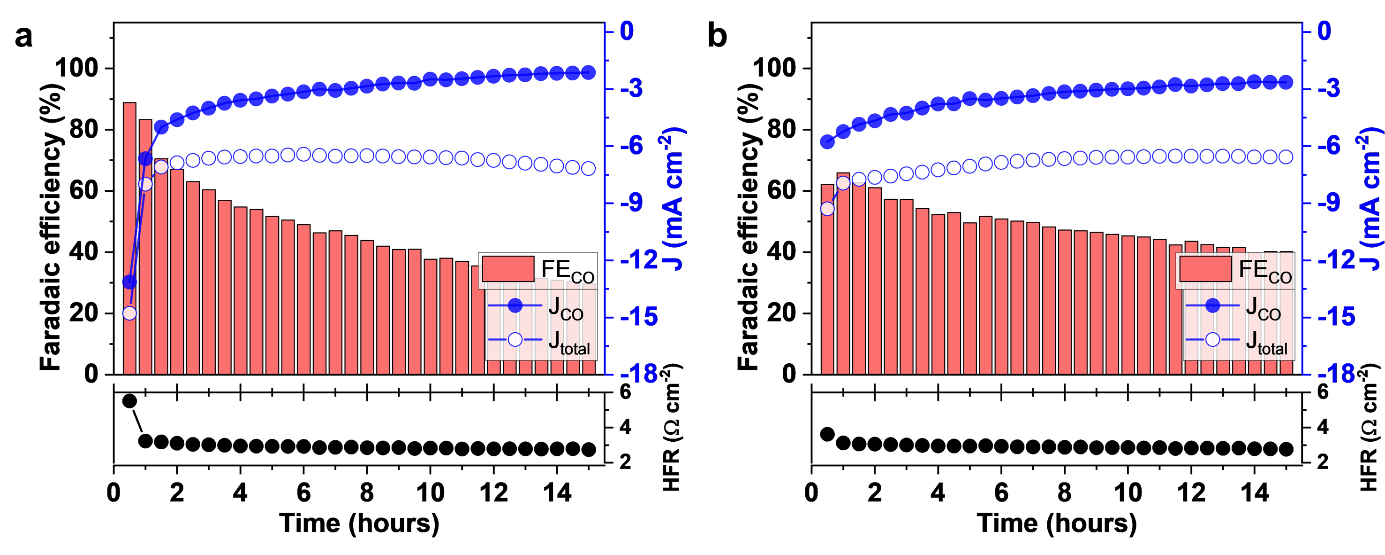
**

**Figure S8.** Durability test of Ni-N-C catalyst in (**a**) Nafion-bonded electrode and (**b**) neutralized Nafion-bonded electrode at ‑1.0 V vs. RHE in 0.5 M KHCO_3_.


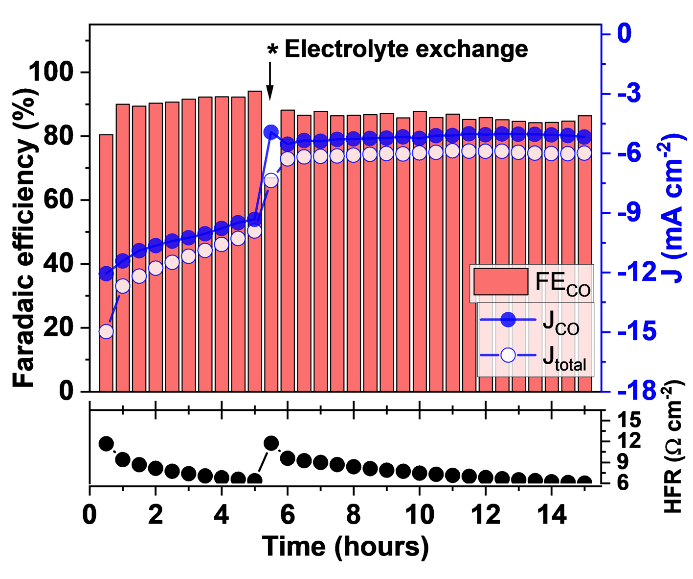


**Figure S9.** Durability test of Ni-N-C catalyst in Nafion-bonded electrode at -0.9 V vs. RHE in 0.1 M KHCO_3_.


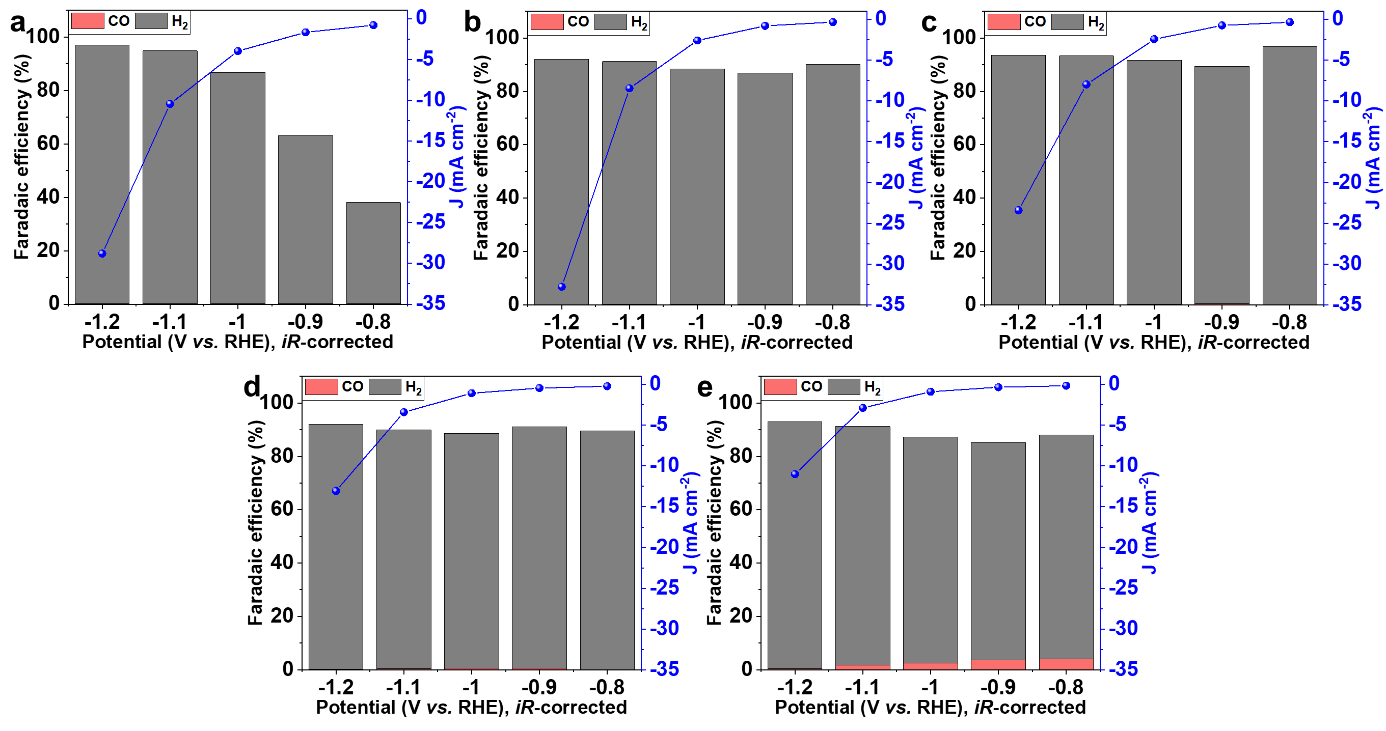


**Figure S10.** CO_2_RR performance in an H-cell of (**a**) SGL 39BB carbon paper without ionomer and with (**b**)  Nafion, (**c**) BPSH-30, (**d**) PiperION, and (**e**) F7N-75 coatings.

**
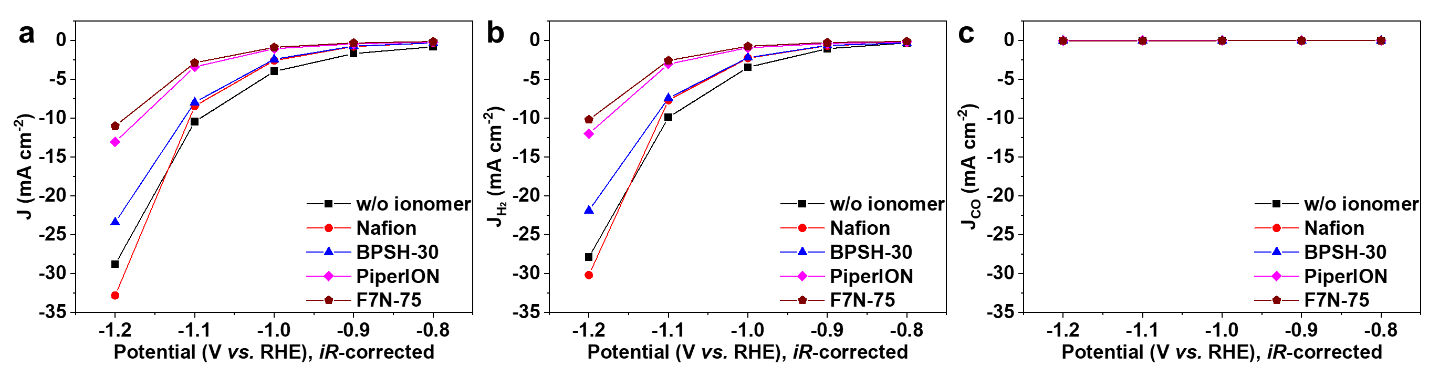
**

**Figure S11.** (**a**) Total current density measured in an H-cell with SGL 39BB carbon paper without ionomer and with different ionomer coatings; (**b**) partial H_2_ current density, and (**c**) partial CO current density.


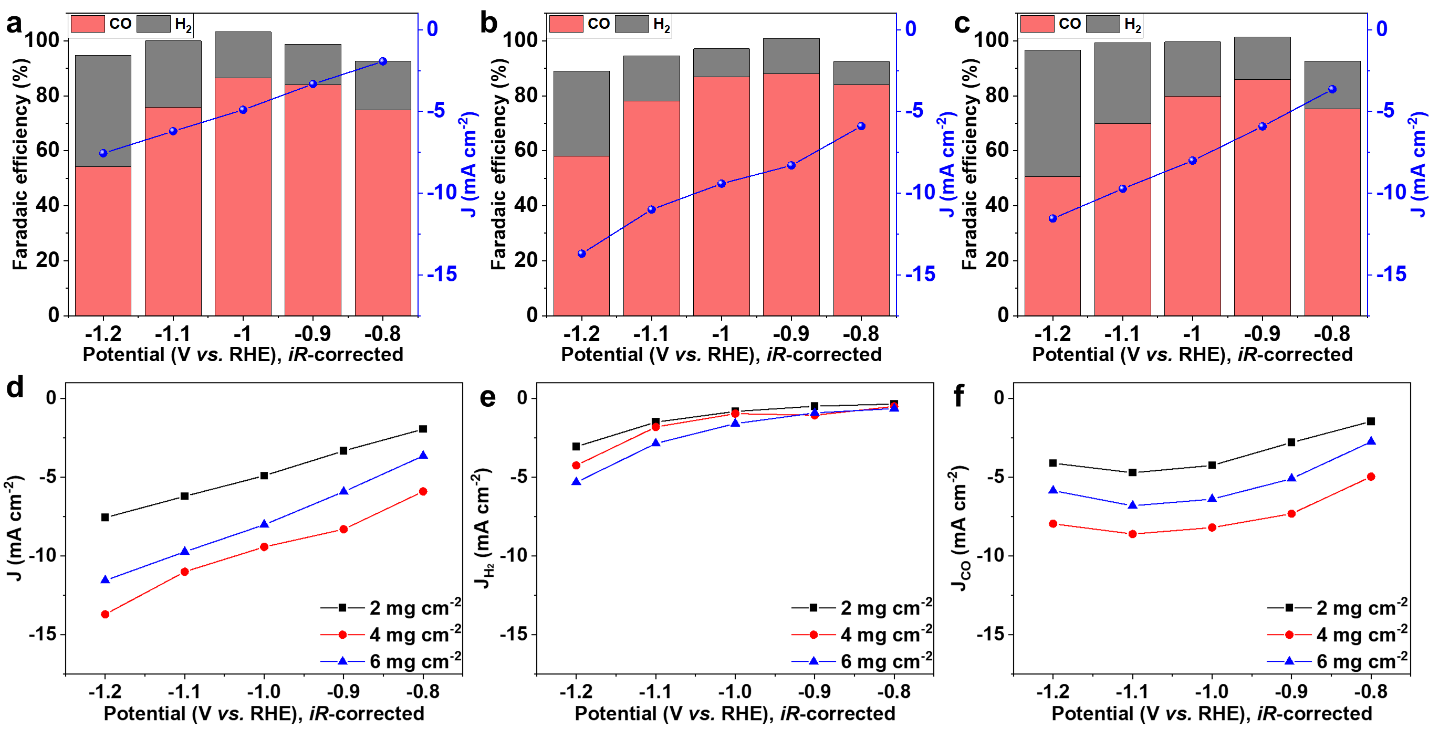


**Figure S12**. Faradaic efficiency and current density measured with Ni-N-C catalyst in an H‑cell using SGL 39BB carbon paper, Nafion ionomer and I/C ratio of 0.3 at different catalyst loadings: (**a**) 2 mg cm^‑2^; (**b**) 4 mg cm^–2^; and (**c**) 6 mg cm^–2^. Current as a function of potential at different catalysts loadings: (**d**) total current density, (**e**) partial H_2_ current density, and (**f**) partial CO current density.


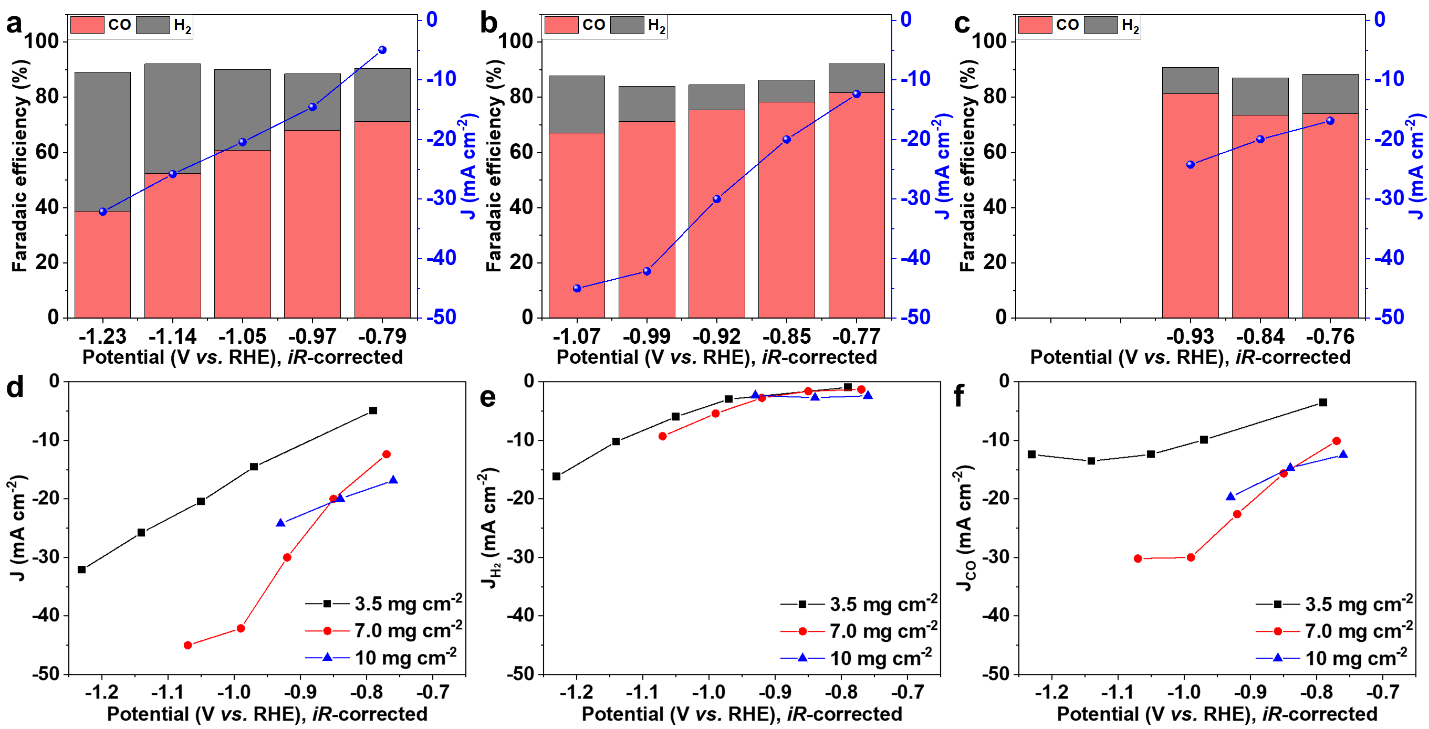


**Figure S13**. Faradaic efficiency and current density measured with Ni-N-C catalyst in a flow cell using SGL 39BB carbon paper, Nafion ionomer and I/C ratio of 0.3 at different catalyst loadings: (**a**) 3.5 mg cm^‑2^; (**b**) 7.0 mg cm^–2^; and (**c**) 10 mg cm^–2^. Current as a function of potential at different catalysts loadings: (**d**) total current density, (**e**) partial H_2_ current density, and (**f**) partial CO current density.


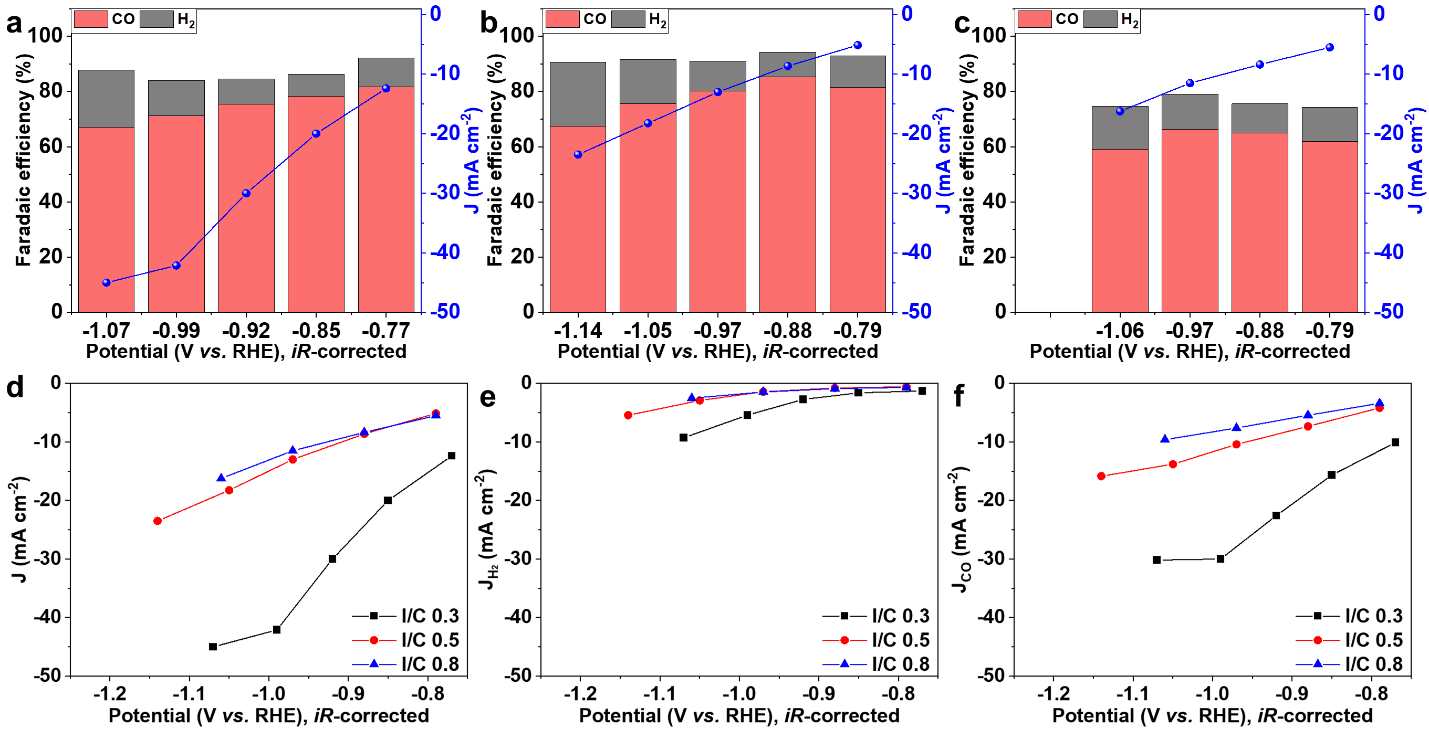


**Figure S14.** Faradaic efficiency and current density measured with Ni-N-C catalyst in a flow cell using SGL 39BB carbon paper, Nafion ionomer, and 7 mg cm^-2^ catalyst loading at different I/C ratios: (**a**) 0.3, (**b**) 0.5, and (**c**) 0.8. Cell performance as a function of potential measured with different I/C ratios: (**d**) total current density, (**e**) partial H_2_ current density, and (**f**) partial CO current density.

**Table S1.** Adsorption energy of molecular fragments from Nafion, BPSH-30, PiperION, and F7N-75 ionomers on the Ni-N-C catalyst.

|  | E_ads_ (eV) | |
| --- | --- | --- |
|  | Horizontal | Vertical |
| Nafion | -0.21 | -0.22 |
| BPSH-30 | -0.81 | -0.53 |
| PiperION | -1.06 | -0.71 |
| F7N-75 | -0.79 | -0.36 |
